# Developmental Restriction of Retrotransposition Activated in *Arabidopsis* by Environmental Stress

**DOI:** 10.1534/genetics.117.300103

**Published:** 2017-08-03

**Authors:** Hervé Gaubert, Diego H. Sanchez, Hajk-Georg Drost, Jerzy Paszkowski

**Affiliations:** The Sainsbury Laboratory, University of Cambridge, CB2 1LR, United Kingdom

**Keywords:** retrotransposition, LTR retrotransposons, *Arabidopsis thaliana*, epigenetic regulation, abiotic stress

## Abstract

Retrotransposons (RTs) can rapidly increase in copy number due to periodic bursts of transposition. Such bursts are mutagenic and thus potentially deleterious. However, certain transposition-induced gain-of-function or regulatory mutations may be of selective advantage. How an optimal balance between these opposing effects arises is not well characterized. Here, we studied transposition bursts of a heat-activated retrotransposon family in *Arabidopsis*. We recorded a high inter and intraplant variation in the number and chromosomal position of new insertions, which usually did not affect plant fertility and were equally well transmitted through male and female gametes, even though 90% of them were within active genes. We found that a highly heterogeneous distribution of these new retroelement copies result from a combination of two mechanisms, of which the first prevents multiple transposition bursts in a given somatic cell lineage that later contributes to differentiation of gametes, and the second restricts the regulatory influence of new insertions toward neighboring chromosomal DNA. As a whole, such regulatory characteristics of this family of RTs ensure its rapid but stepwise accumulation in plant populations experiencing transposition bursts accompanied by high diversity of chromosomal sites harboring new RT insertions.

TRANSPOSABLE elements (TEs) are an integral part of most prokaryotic and eukaryotic genomes. They are mostly transcriptionally silent and their transgenerational transmission uses host mechanisms propagating chromosomal DNA. However, sporadic TE-specific replication/transposition cycles increase their copy number through new chromosomal insertions. Past bursts of such TE activity can be studied and dated according to the degree of divergence of TE sequences due to gradual accumulation of mutations (Wicker and Keller 2007). It is evident that historical bursts of transposition have shaped chromosomal architecture and contributed to various activities of the genome (Lisch 2013). However, studies of ancient transpositions cannot reveal the physiological and environmental circumstances under which the bursts occurred and how these were controlled.

Transposition of the two main classes of TEs, retrotransposons (RTs) and DNA transposons, is by “copy-paste” and “cut-paste” mechanisms, respectively. Copy-paste involves transposon transcription, RNA-directed synthesis of extrachromosomal DNA (ecDNA), and subsequent chromosomal integration (Lisch 2009). Due to this replicative mode of transposition, RTs rapidly increase in copy number. As a consequence, this class of TEs is very abundant and widely distributed in nature, especially in plants (Sabot and Schulman 2006). Of the plant RTs, the most plentiful are a subclass with long terminal repeats (LTR) marking the ends. For example, >85% of the maize genome is comprised of TEs, of which over 75% are RTs that can be classified mostly as LTR-containing retrotransposons (LTR_RTs) (Tenaillon *et al.* 2010).

Despite the abundance of plant LTR_RTs, and of well-documented historical transposition bursts (Wicker and Keller 2007), only a few LTR_RT movements have been recorded in real time (Grandbastien *et al.* 1989; Hirochika *et al.* 1996; Mirouze *et al.* 2009; Tsukahara *et al.* 2009; Ito *et al.* 2011), and these mostly due to purely serendipitous detection of retrotransposition through forward mutations resulting from new RT insertions. Optimally, these should cause phenotypic changes that can be subsequently characterized at the molecular level (Miyao *et al.* 2007; Piffanelli *et al.* 2007; Lisch 2013). Thus, studies of LTR_RT activities are often restricted to the detection of transcripts or extrachromosomal DNA copies (Cavrak *et al.* 2014).

It has been reported recently that the retrotransposition cycles of LTR_RTs in *Arabidopsis* and in rice are controlled by particular host mechanisms. Notably, rice retrotransposon *Tos17* initially induced for transposition by tissue culture (Hirochika *et al.* 1996), is suppressed *in planta* by DNA methylation (Cheng *et al.* 2015). It has been revealed in *Arabidopsis* that particular phases of the LTR_RT life cycle can be linked to specific epigenetic, developmental, or environmental factors that interfere with LTR_RT transcription, reverse-transcription, and/or chromosomal integration. This amounts to regulation at the transcriptional and post-transcriptional levels specific for a particular LTR_RT family (Mirouze *et al.* 2009; Ito *et al.* 2011). For example, the transcription of LTR_RT *Evadé* is restricted by DNA methylation, and can occur only when the *Evadé* locus becomes hypomethylated. The synthesis of *Evadé* ecDNA is limited by the activity of histone methyltransferase, which dimethylates histone 3 at lysine 9 (H3K9me2), and/or by the activity of the plant-specific RNA polymerase IV involved in the biogenesis of small RNAs (Mirouze *et al.* 2009). Once the ecDNA of *Evadé* is synthesized, it is still the subject of activity restricting its chromosomal integration; this originates from the maternal parent but is not well understood (Reinders *et al.* 2013).

Very different to *Evadé* is the regulation of the life cycle of the related LTR_RT *Onsen* (Ito *et al.* 2011). *Onsen* is activated by elevated temperature in both wild-type *Arabidopsis* and in mutants deficient in the biogenesis of small RNAs. In both types of plants, *Onsen* synthesizes its ecDNA but transposition occurs only in heat-stressed plants deficient in the biogenesis of small RNAs, and not in the stressed wild type. Moreover, plants stressed as small seedlings instantly accumulate ecDNA of *Onsen*, which decays during subsequent growth, and seems to be at a very low level at flowering when *Onsen* transposition takes place (Ito *et al.* 2011). This implies developmental propagation of stress memory in plants deficient in small RNAs until they reach a specific developmental window permissive for *Onsen* retrotransposition (Ito *et al.* 2011). Such multi-level and transposon-specific regulation of LTR_RT life cycles suggests that extrapolation of regulatory mechanisms, from the observed efficiencies of initial steps, such as transcription and reverse-transcription, to the final step of LTR_RT transposition, may be superficial and potentially misleading.

Here, we provide insights into previously overlooked regulatory steps governing *Onsen* retrotransposition. We have observed that *Onsen* movement is constrained to single bursts that result in multiple new insertions in a given lineage of germline progenitor cells. Once such a retrotransposition burst occurs, subsequent secondary transposition bursts in progenitor cells appear to be inhibited. This unexpected retrotransposon self-restriction, or possible developmental inhibitory effects of the host, generates a high degree of intra and interplant variation in the chromosomal distribution of new transposon insertions and limits the number of new transposon copies per individual progeny plant in a given plant population. In addition, the new transposon insertions seem to have mostly short distance regulatory influences toward neighboring host genes, and these influences are largely dependent on the arrangement of the pre-existing transcriptional units at the insertion sites.

## Materials and Methods

### Plant material and experimental conditions

Seeds were surfaced-sterilized and sown in Petri dishes with agarified (0.8% Bacto Agar, Becton Dickinson) 0.5× MS medium (Duchefa), containing 1% sucrose and 0.05% MES at pH 5.7. After stratification for 3 days at 4°, plants were grown in a CU-22L growth chamber (Percival) under strict temperature control and a 12/12 hr (day/night) light cycle. Typically, 7-day-old plants grown at 21° were used and treated further at different temperatures according to the experimental design described below. For the heat-stress-induction of *Onsen* transposition in *nrpd1-3*, a prechilling step was performed to increase the relative activation. Seedlings were first placed at 4° for 24 hr on a built-in chilling platform within the growth chamber (controlled by an external mini-chiller), but with the chamber temperature set to 6°. This chilling pretreatment was followed by 24 hr with the chamber set to 37° for the heat-stressed plants; control plants were moved to 21°. The same experimental design was applied for the transcriptome experiments, where pools of ∼50 progeny seedlings from two independent biological replicates of *nrpd1-3* and *nrpd1-3* plant 2L were collected directly after heat-stress or control treatments.

### Molecular biology

Total RNA and DNA were isolated using the Plant-RNeasy kit (Invitrogen) and the DNeasy Plant Mini Kit (Qiagen), respectively. Transposon display was performed as described previously (Ito *et al.* 2011) based on the Genome Walker Universal kit (Clontech). Briefly, genomic DNA was digested with *Dra*I restriction enzyme and purified with the QIAquick DNA Purification Kit (Qiagen). DNA was ligated with genome walker adaptors, diluted, and used as template for PCR reactions carried out using a primer specific for the adaptor (GenWalk_AP1) and a primer specific for *Onsen* (ONS_312). PCR conditions were 5 min at 95° followed by 33 cycles of 30 sec at 94°, 30 sec at 58°, 1 min at 72°; and a final elongation. A list of primers used is available in Supplemental Material, Table S4.

### Next-generation sequencing (NGS)

For RNAseq analyses, 50–70 seedlings (per biological repeat) were pooled for RNA extraction. Strand-specific libraries were prepared with 2 µg of RNA using the TruSeq Stranded mRNA Sample Prep Kit (Illumina), following the provider’s instructions. For whole-genome-resequencing, at least five plants arising from seeds of the same silique of heat-induced *nrpd1-3* mutant plants were pooled and DNA-extracted. Library preparation used 1 µg of DNA with the TruSeq DNA PCR-Free Library Prep kit (Illumina), following the provider’s instructions. NGS was performed with a Next-Sequation 500 (Illumina) platform reporting 150-bp and 75-bp paired-end reads for RNAseq and DNAseq, respectively. Each sample was resequenced to an average depth of at least 17-fold. Analysis of sequencing data were conducted with standard open-source software. Trimming was performed with Trimmomatic (Bolger *et al.* 2014), applying ILLUMINACLIP parameters:2:10:5:1. For transcript level analysis, reads were mapped with TopHat (Trapnell *et al.* 2009) on the *Arabidopsis* TAIR10 assembly (www.arabidopsis.org); using parameters–max-multihits 1–read-realign-edit-dist 0–no-mixed. Mapped reads were subsequently counted using htseq-count (Anders *et al.* 2015) with parameters–order name–type = exon–stranded = no. We applied a stringent presence-call filter, restricting the analysis to those annotated genes with more than five counts-per-million in at least two biological replicates. Differential expression was assessed with edgeR (Robinson *et al.* 2010), using as thresholds 1 Log2 fold change and a Benjamini-Hochberg’s FDR < 0.05. Bowtie2 (Langmead and Salzberg 2012) was employed for mapping DNA sequencing reads with parameters–very-sensitive -X 1000–non-deterministic. Further handling of NGS reads was carried out with SAMtools (Li *et al.* 2009) and Picard (http://picard.sourceforge.net.). Custom-made workflows, available at https://github.com/diegohernansanchez/, were developed inhouse for efficient detection of new *Onsen* insertions, using SAMtools (Li *et al.* 2009), BEDtools (Quinlan and Hall 2010), and Python scripts (www.python.org). We applied a combined strategy based on finding discordant paired-end reads and junction reads around insertion points. In a first step, pair-end reads were first mapped on an *Onsen*-masked genome, recovering those in which one mate mapped to a chromosomal location but the other remained unmapped. We then filtered for those in which the second mate mapped to *Onsen*’s LTR. In a second step, we recovered all junction reads between the genome and *Onsen*, by recognizing unmapped reads blasting to *Onsen*’s LTR 5′ and 3′ extremities and trimming away the transposon sequence. We remapped these short sequences using ‘soft clipped’ Bowtie2 mapping, thus accounting for tandem-site-duplications, with parameters–local–very-sensitive-local–score-min L,5,0–np 0. Chromosomal mapped reads from the first and second steps accumulated around *Onsen* members and putative new insertions. Finally, manual assessment was used to confidently define new insertion’s genomic coordinates, ruling out false positives by comparing sequenced *nrpd1-3* and wild-type backgrounds. For validation, we selected 21 individual new insertions, and, in all cases, they were corroborated by standard PCR in genomic DNA (Figure S2 in File S1). A list of primers used is available in Table S4. Raw data were deposited in ArrayExpress (www.ebi.ac.uk/arrayexpress/) under accession numbers E-MTAB-5883 and E-MTAB-5884; and materials are available upon request.

### Statistical assessment of chromosomal distribution for *Onsen* new insertions

To statistically assess whether new *Onsen* insertions are randomly distributed, we first divided the genome into euchromatic and heterochromatic regions, defined using H3K9me2 methylation data obtained from GSE37075 (Deleris *et al.* 2012), and visualized the enrichment value per 60 bp tile for each chromosome. In a next step, we applied smoothing as moving average with 100 tiles period and filtered for regions above the moving average trend line of 1.5 (conservative threshold), rounding the coordinates to closest 100 kbp. Next, we randomly sampled 338 loci with the same lengths and same chromosomal locations as the 338 new *Onsen* insertions, and counted, for each 1 Mbp sliding window (focusing on euchromatin with 332 insertions), how frequently they appeared across sliding windows (Figure S3 in File S1). To statistically test the variance of insertion counts we also randomly sampled 332 loci in 1000 independent permutation runs. A gamma distribution was fitted to the histogram of these 1000 variances of randomly sampled loci, and moment matching estimators were used to estimate the shape and scale parameters of the gamma distribution. We used this gamma distribution to report the probability of exceeding the observed variance of real *Onsen* insertions as *P*-value (shown in Figure S3 in File S1). Computationally reproducible scripts for these analyses can be found at https://github.com/HajkD/.

### Data availability

The authors state that all data necessary for confirming the conclusions presented in the article are represented fully within the article.

## Results

We previously documented efficient retrotransposition of *Onsen* in flowers of *nrpd1* plants heat-stressed as 7-day-old seedlings (Ito *et al.* 2011). Multiple new insertions of *Onsen* were reproducibly recorded in the progeny of these plants. Remarkably, the number and distribution of new insertions differed not only between different plants but also between different flowers of the same plant (Ito *et al.* 2011). These results suggested the existence of a particular developmental window during flowering of plants defective in siRNA in which heat-stress-activated *Onsen* transposes. Moreover, new *Onsen* insertions are well transmitted to the progeny, thus they persist during female and/or male meiosis, the development of at least one of the gametophytes, and during fertilization and embryogenesis.

**Figure 1:**
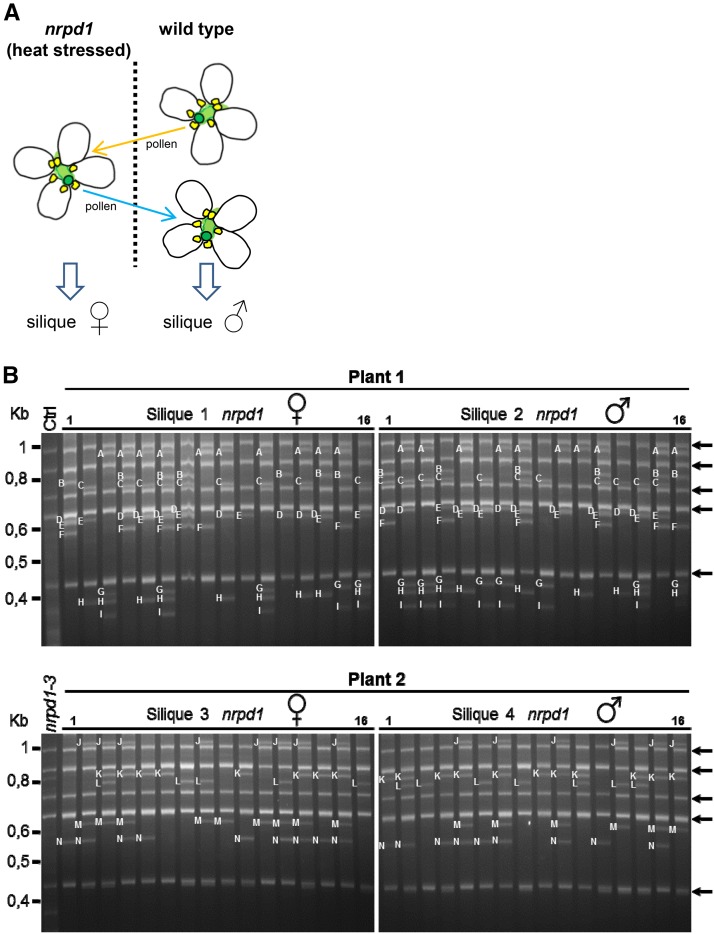
Reciprocal crosses using single flowers from heat-treated *nrpd1* plants. (A) Schematic of the experimental design in which heat-treated *nrpd1* plants were used as pollen acceptor (♀: orange arrow) or pollen donor (♂: blue arrow). Corresponding siliques were harvested from two plants. (B) Transposon display of the progeny from reciprocal crosses of two parental plants (plant 1 and plant 2). Black arrows mark the bands from *Onsen* chromosomal copies originally present in the Columbia accession (Ctrol lane). Related neo-insertions detected in siblings are indicated by the same letters.

To examine in more detail possible regulatory mechanisms associated with *Onsen* transposition, we asked whether transposition competence and the efficiency of transgenerational transmission of newly inserted copies of *Onsen* differ between male and female germlines. For example, it is possible that paternal and maternal germ cell lineages differ in permissiveness for *Onsen* movement and/or the transgenerational propagation of new insertions. In this case, it would be expected that reciprocal crosses of the same flower would result in different patterns of insertions. To examine whether or not patterns of *Onsen* insertion are affected by the parent of origin, we performed transposon display of the progeny from reciprocal back-crossing of single flowers of *nrpd1* plants subjected to heat stress (Figure 1A). As revealed by transposon displays, new *Onsen* insertions were equally present and inherited through heat-treated *nrpd1* paternal and maternal gametes, regardless of the direction of the cross (Figure 1B). Thus, there was no indication of parental influence on the frequency of *Onsen* transposition or transgenerational transmission of new insertions.

**Figure 2:**
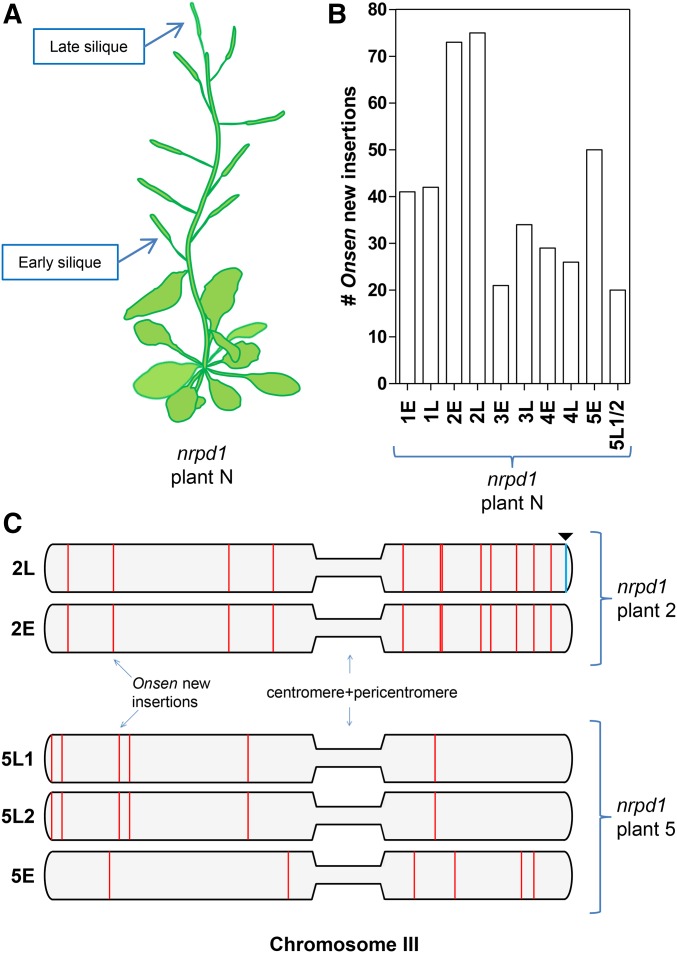
Developmental restriction of *Onsen* retrotransposition. (A) Schematics of the experimental design: progeny of heat-treated *nrpd1* plants from “early” or “late” siliques were screened for new *Onsen* insertions by whole genome sequencing. (B) Interplant variation in number of new insertions. The count of new *Onsen* insertions is shown for progeny of both “early” (E) and “late” (L) siliques in all five heat-treated *nrpd1* lines analyzed. (C) Schematic representation of chromosome III of *nrpd1* plant 2 and *nrpd1* plant 5 progeny (E, early silique; L, late siliques) with new *Onsen* insertions (marked as red vertical lines). Note that for *nrpd1* plant 2, all new insertions in 2E-2L are identical, but silique 2L acquired two additional insertions (one showed here for chromosome III as blue vertical line with a triangle), which is consistent with a single retrotransposition burst in the progenitor cell lineage of the two flowers. In *nrpd1* plant 5, patterns of new insertions differ between early and late siliques (5E/5L1 and 5L2), suggesting two independent retrotransposition bursts; the two late siliques 5L1 and 5L2 share identical new insertions, consistent with a single retrotransposition in the progenitor cell lineage of the two late flowers. These data are consistent with two developmental windows for transpositional competency.

Next, in an attempt to identify a possible developmental window permitting transposition, we examined whether the developmental position of flowers influences the efficiency and pattern of new *Onsen* insertions. Seeds from the earliest and latest emerging siliques (*i.e.*, derived from the first and the last flower developing on the same flowering stem) of five heat-stressed *nrpd1* plants (Figure 2A) were harvested separately. In addition, from one of the plants we also harvested two neighboring late-emerging siliques (*nrpd1* plant 5). We isolated DNA from five progeny seedlings representing each silique (in total 11 siliques). Using whole-genome sequencing, we determined the number and positions of all new *Onsen* insertions in this material, a total of 338. To validate the genomic positions of insertions arising from the whole genome sequencing, we performed PCR for randomly chosen neo-insertions using genomic DNA. In all cases, this confirmed the presence of a newly inserted copy of *Onsen* (Figure S1 in File S1).

We observed that number of new insertions significantly varied between progenies of five parental plants, and ranged from 27 to 75 new *Onsen* insertions (Figure 2B). Since all the parental plants were grown and heat-stress-treated in parallel, such variation in the number of new insertion indicates highly variable permissiveness of different individuals for *Onsen* transposition. However, the new insertions were uniformly distributed between all five chromosomes, varying only between 2.6 and 3.2 insertions per Mb, an average of 2.8 new insertions per Mb (Table 1). This is consistent with the absence of “hot” or “cold” chromosomal areas for *Onsen* insertion (Figure S2 in File S1 and Table 1). However, when we partitioned the *Arabidopsis* genome into euchromatin and heterochromatin, we found that 332 *Onsen* insertions were present in 109 Mbp of euchromatin and only 6 in 16 Mbp of heterochromatin, which is a statistically significant enrichment of *Onsen* insertions in euchromatic regions (*P* = 5.9e−06; Fisher’s exact test for count data). We also found that *Onsen* insertions in euchromatic chromosomal regions have a larger distribution variance than would be expected for random insertions (*P* = 1.012e−06; permutation test; Figure S3 in File S1). Therefore, possible preferences of particular chromosomal targeting in euchromatin cannot be excluded, and only future analyses of many more new *Onsen* insertions could resolve this issue.

**Table 1:** Number of independent *Onsen* neo-insertions found in each line and chromosome from “early-late” siliques experiment

	Chr. I	Chr. II	Chr. III	Chr. IV	Chr. V	Total
1E	12	5	7	8	9	41
1L	9	4	8	7	14	42
2E-2L (common)	23	11	12	15	12	73
2L-specific	1		1			2
3E	6	3	2	5	5	21
3L	7	8	8	5	6	34
4E	7	5	7	5	5	29
4L	7	5	6	4	4	26
5E	13	15	6	6	10	50
5L1-5L2	3	2	6	4	5	20
Total	88	58	63	59	70	338
Neo-insertions/Mb	2.9	2.9	2.7	3.2	2.6	2.8

Importantly, there was a very strong tendency for insertions into genes (almost 90% of new insertions). We performed an enrichment analysis for count data based on hypergeometric distributions, and confirmed that *Onsen* neo-insertions are significantly over-represented in gene space compared to TEs or intergenic spaces (*P* < 2.2e−16). Moreover, 80% of new insertions were mapped to transcribed regions of genes (Table 2); > 60% were found in exons (Table 2). These findings suggest that chromatin properties of genic regions and/or the presence of processed transcripts may attract reinsertion of *Onsen*. Interestingly, new insertions are found relatively often in heat stress responsive genes as evaluated by transcriptome analysis (see RNAseq profiles below). From 276 protein-coding-genes where *Onsen* inserted in the *nrpd1* background, transcripts for ∼47% were detected in *nrpd1* seedlings, and ∼33% were heat-responsive (either up or downregulated compared to control condition, with ≥ 1 Log2 fold change and FDR < 0.05, Table S1). Although standard ontological examination of genes attracting *Onsen* suggested preferential insertions into genes related to defense responses (*P* < 0.01) (Table S2), when this apparent enrichment was corrected for the gene lengths with *Onsen* insertions, its significance became insignificant (*P* = 0.5058; Pearson’s Chi-squared test).

**Table 2:** Genic and intergenic location of *Onsen* neo-insertions from “early-late” silique experiment

	500 bp < Promoter Region < 1000 bp	Promoter Region <500 bp	5′UTR	Exon	Intron	3′UTR	Intergenic >1000 bp	TE
Number of insertions	9	17	11	211	42	12	19	17
Proportion of neo-insertions in each domain (%)	2.7	5.0	3.3	62.4	12.4	3.6	5.6	5.0
			81.7			
		86.7				
	89.3					

Intriguing patterns were observed in the distribution of new insertions between early and late siliques of the same plant. In the majority of cases (four out of five), the distributions of new insertions in early and late siliques were entirely different (*nrpd1* plants 1, 3, 4 and 5; Figure S2 in File S1). This is consistent with previous observations suggesting that transposition bursts take place in different flowers independently (Ito *et al.* 2011). Notably, we recorded almost identical distribution (two additional reintegrations in the late silique) of new *Onsen* insertions in early and late siliques of *nrpd1* plant 2, and in the two late siliques of *nrpd1* plant 5, respectively (Figure 2C, Figure S2 in File S1, and Table 1).

These patterns suggested that, in these cases, transposition bursts occurred prior to differentiation of the two flowers. Therefore, the developmental window permissive for transposition bursts could be extended to include both “flower-specific” and “preflower” bursts. It is remarkable that we did not observe additive patterns indicative of “flower-specific” or “preflower” bursts occurring consecutively in the same cell lineages, as would be expected given the two sequential developmental windows permissive for transposition. The observed exclusivity of one of the two developmental windows suggests that when a “preflower” burst occurs, this inhibits a later additional “flower-specific” burst. In other words, it appears that a retrotransposition burst inhibits subsequent bursts in the same cell lineages, which are germline progenitors. However, when the cell lineages are separated (*e.g.*, to form different flowers) prior to transposition burst, independent bursts of retroelement movement can occur in each of them. This observation would imply some type of feedback inhibition of *Onsen* retrotransposition bursts, restricting retrotransposition to only one burst that is passed to the progeny, although isolated individual insertions may still occur (*nrpd1* plant 2; Figure S2 in File S1 and Table 1). However, it cannot be excluded that independent bursts in several flower progenitor cells of the inflorescence meristem could also contribute to the observed variability in chromosomal distribution of insertions documented for different flowers.

**Figure 3:**
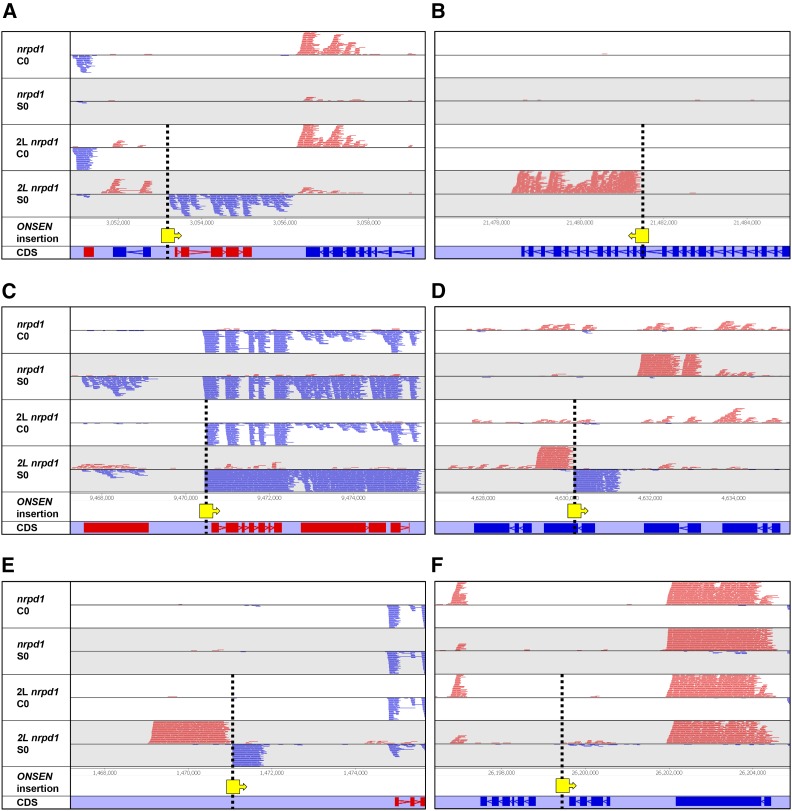
Heat-induced transcriptional activation of chromosomal regions adjacent to new *Onsen* insertions. Strand-specific RNAseq analysis of pooled *nrpd1* progeny and plant 2L progeny (Table 1) subjected to heat stress (S0) or grown in control conditions (C0). The figure displays representative screen shots of genome browser. New *Onsen* insertions in plant 2L are depicted as yellow boxes, with arrows indicating the direction of insertion. (A) Insertion into a gene promoter in sense orientation in respect to the affected gene. (B) Insertion into coding region of a gene in sense orientation. (C) Insertion into a gene promoter in sense orientation (note transcriptional activation of the neighboring gene). (D) Insertion into coding region of a gene in antisense orientation. (E) Insertion into intergenic region. (F) Insertion between genes showing negligible transcriptional disturbance. Treatments and genotype given left to the chromosomal tracks; CDS track mark gene coding sequences, red features represent transcripts in left to right orientation, and blue features transcript from right to left.

It was documented previously that genes adjacent to new insertions of *Onsen* acquire transcriptional response to heat stress (Ito *et al.* 2011). However, it was not clear how far from insertions the influence of an *Onsen* prevails, and to which extent genomic features neighboring new *Onsen* insertions would modulate its regulatory impacts. Therefore, we determined heat-stress-induced transcript levels of chromosomal sequences flanking new insertions. For that we performed transcriptional profiling (RNAseq) of control and heat-stressed plants of *nrpd1* plant 2L, for which we mapped 75 new insertions (Figure 3 and Table 1). The transcript levels were compared to the same region in the original *nrpd1* mutant that lacked insertions (Figure 3 and Figure S5 in File S1).

Insertions were found in both sense and antisense orientations in relation to the transcripts of affected genes. New *Onsen* copies were located in gene promoters or in gene bodies (in introns or exons) and rarely in intergenic regions (Table 2). In general, transcriptional activation was detected at relatively short distances from the new *Onsen* copies, and these distances were largely influenced by genic environment; therefore, it is impossible to provide general regulatory rules across genic and intergenic insertions based on any kind of meta-analysis of this data set. However, typical regulatory influences for certain types of chromosomal environment can illustrate the regulatory influences of new *Onsen* insertions and are presented on Figure 3. For example, sense insertions in gene promotors or gene bodies often activated transcription of the target gene, and the activation distance followed the length of a native gene transcript (Figure 3, A and B). This pattern was found in 61 genes targeted by *Onsen*, of which 56 were deemed expressed, and 46 (∼82%) showed activated transcript levels between *nrpd1* plant 2L and *nrpd1* upon heat stress (Table S3). Similarly, out of seven *Onsen* insertions in promoters of genes, of which five were deemed expressed, all five were activated upon heat stress in *nrpd1* plant 2L (Table S3). Interestingly, if the target gene was linked to another gene in the same orientation, *Onsen*-mediated activation sometimes seemed to expand over the adjacent gene (Figure 3C). To assess these transcriptional alterations, we investigated the transcriptomic data for 74 of these adjacent genes deemed expressed. Out of these, 30 (∼41%) showed altered transcript levels in *nrpd1* plant 2L subjected to heat stress (Table S3). We calculated the frequency distribution of distances between new insertions of *Onsen* and the genes transcriptionally affected (30) and not affected (44); no significant differences between the two groups were observed (Kolmogorov-Smirnov test, *P* = 0.4833, Figure S4 in File S1). Therefore, it cannot be predicted whether transcript level of an active gene neighboring a new *Onsen* insertion will be altered or not. However, when altered, the typical *Onsen* influence occurs within a small distance of ∼2 kb; with occasional exceptions of longer distances that may reach even 10–12 kb (Figure S4 in File S1).

In case of antisense genic insertions, transcriptional activation in 81% the cases seemed to be bidirectional (Figure 3D and Figure S5 in File S1). This effect is similar to transcriptional activation by *Onsen* insertions found in intergenic regions (Figure 3E). We also found rare examples of absence or negligible transcriptional alterations following *Onsen* insertions (Figure 3F and Figure S5 in File S1). Thus, *Onsen* seems not to have long-distance transcriptional enhancer-like effects, but predominantly a rather local influence on genes directly affected by the insertions, and less often on those being in their vicinity. Noticeably, for *Onsen* insertions inducing heat-stress-triggered transcription in the same direction as the transcription of interrupted genes, the induced transcripts seem to terminate in the region where gene transcripts would normally terminate. In contrast, insertions of *Onsen* inducing transcripts in an antisense direction in relation to the gene transcription resulted in bidirectional transcripts mostly terminated in close proximity to the insertion. To fully illustrate the variable influence of chromosomal environment on regulatory impacts of new *Onsen* insertions, we provide screenshots for all 75 regions acquiring new *Onsen* copies (Figure S5 in File S1).

## Discussion

It seems that, for two plants tested by reciprocal cross of a single flower, new *Onsen* insertions are transmitted with equal efficiency through male and female gametes, suggesting that, in these cases, the observed bursts of transposition had a limited impact on the gametophytic developmental cycle and embryogenesis. This could be the result of preferential targeting of new insertions and/or the activation of mechanisms restricting uncontrolled proliferation of *Onsen* copies. Indeed, we observed favored targeting of *Onsen* into genes; however, this is inconsistent with the low impact on the transgenerational transmission of new *Onsen* insertions. On the other hand, the frequent targeting of genes involved in particular processes not directly affecting plant survival, such as plant defense, may explain in part why new insertions do not interfere with the plant sexual cycle, and, thus, with their transgenerational inheritance. Interestingly, genic preference of insertion has been also documented for *Tos17*, and, also in this case, genes related to plant defense have been preferentially targeted (Miyao *et al.* 2003).

It appears that *Onsen* activates only a single burst of transposition per plant generation in a given lineage of germline progenitor cells. Importantly, it has been shown that progeny of stressed *nrpd1* plants with new insertions can again undergo additional *Onsen* transposition bursts (Matsunaga *et al.* 2015). Therefore, increased *Onsen* copy number is not the trigger for its transpositional inactivation, as documented for *Evadé* (Mari-Ordonez *et al.* 2013). Alternatively, mechanisms restricting retrotransposition might be encoded by the retroelement itself, as recently documented for the yeast *Ty1* retrotransposon (Tucker *et al.* 2015), or a change in the competence of particular cell lineages experiencing *Onsen* transposition precludes subsequent transposition bursts. Notably, this switch of competence occurs only within the same plant generation, and is reset between generations (Matsunaga *et al.* 2015).

The late developmental timing of transposition bursts in combination with just single independent bursts in a given cell lineage increases immensely the variation in patterns of new insertions in progeny of the transposition-competent parents. In addition, since transposition bursts occur predominantly during development of flowers, and prior to separation of the male and female germlines, multiple progeny derived from such flowers will have similar patterns of insertions segregating randomly as homozygous or hemizygous. This results in a related insertion patterns in multiple offspring; deleterious insertions are exposed to selection as homozygotes, but may persist and be further transmitted in the hemizygous state.

The observed high variability in chromosomal position and number of *Onsen* new insertions among the progeny of different plants, despite parallel, and thus identical, heat stress and growth conditions, may lead to enhancement of the proliferation of retrotransposons. High variability in the distribution of newly inserted copies results in a certain number of neutral or near-neutral new insertions, which increases the possibility of their inheritance over multiple generations. In this regard, it is important that, as observed, most of the regulatory influence of relocated *Onsen* is local, or at relatively short distances from the insertion sites. This is in contrast to some maize transposons, whose regulatory influence extends to distances of tens of thousands of bases (Studer *et al.* 2011). Such limitation of *Onsen* impact, hugely influenced by the chromosomal transcriptional landscape, likely limits alterations in the host’s gene regulatory networks and, thus, potentially mitigates negative effects. This may further enhance the chance of transgenerational transmission of novel *Onsen* insertions.

Moreover, high inter and intraplant variation in the number of new insertions, their distribution, and their allelic state (homo or hemizygous) should result in a rapid increase in *Onsen*-generated genetic diversity, which is then the subject of natural selection. Indeed, transposable elements are known to contribute to genome evolution through myriad mechanisms, such as the generation of novel regulatory elements (*e.g.*, promoters, enhancers, or silencers), and contributions to chromosomal recombination and the epigenetic landscape (Lisch 2009). Although the mechanisms described here that achieve high variability in new insertions, and restrict their copy number and negative regulatory impacts, might be specific only to *Onsen*, it is conceivable that related strategies could be exploited also by other retrotransposons.

## Supplementary Material

Supplemental material is available online at www.genetics.org/lookup/suppl/doi:10.1534/genetics.117.300103/-/DC1.
