# Supplementary material for: Developmental Restriction of Retrotransposition Activated in Arabidopsis by Environmental Stress
Source: Genetics. 2017 Aug 3;207(2):813–21. doi: 10.1534/genetics.117.300103 (PMC5629341; doi:10.1534/genetics.117.300103)
Supplement: Supplementary file 2 [file 813TableS2.pptx]

## Slide 1
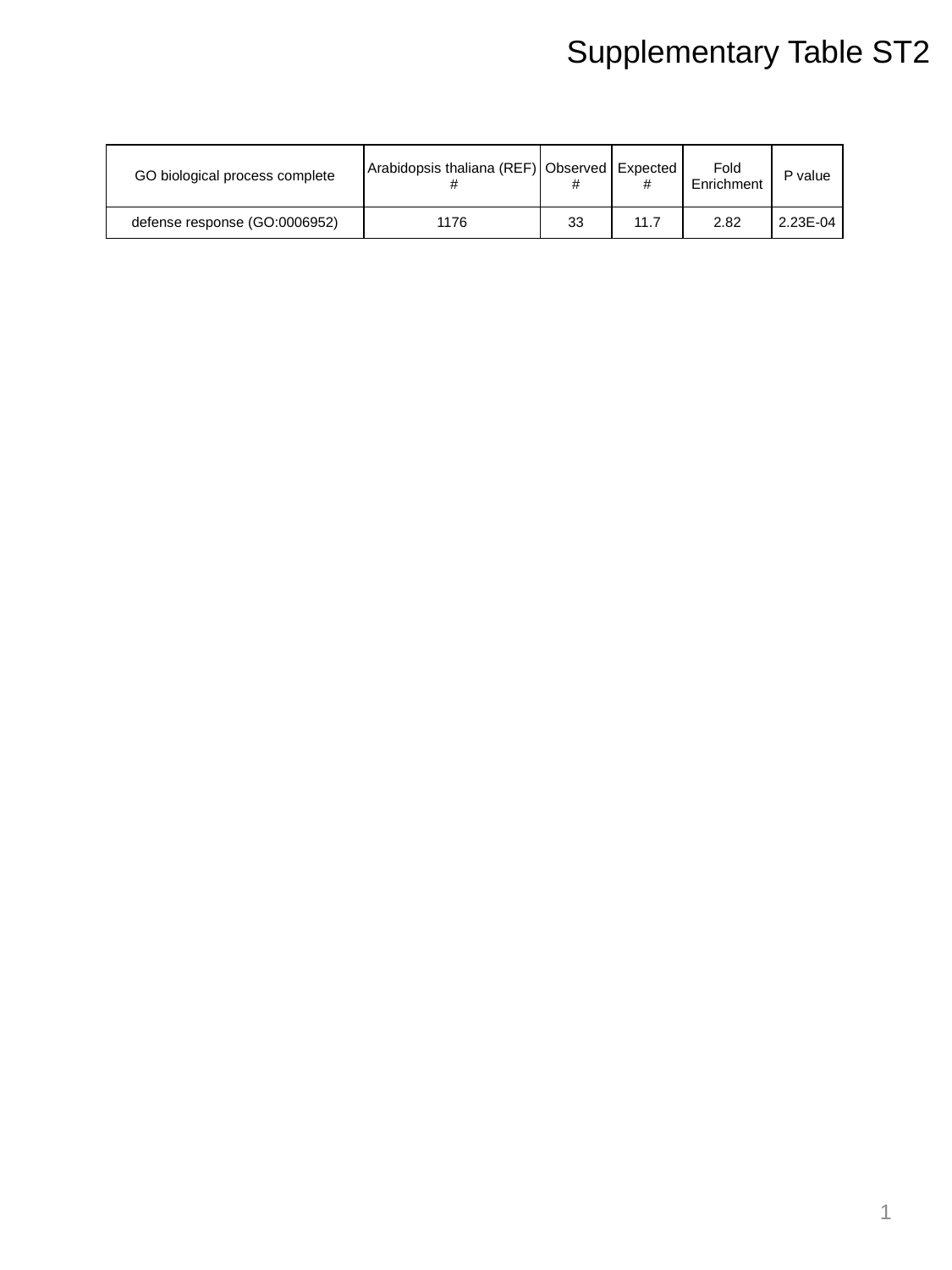

Supplementary Table ST2
| GO biological process complete | Arabidopsis thaliana (REF) # | Observed # | Expected # | Fold Enrichment | P value |
| --- | --- | --- | --- | --- | --- |
| defense response (GO:0006952) | 1176 | 33 | 11.7 | 2.82 | 2.23E-04 |
1
