# Supplementary material for: Developmental Restriction of Retrotransposition Activated in Arabidopsis by Environmental Stress
Source: Genetics. 2017 Aug 3;207(2):813–21. doi: 10.1534/genetics.117.300103 (PMC5629341; doi:10.1534/genetics.117.300103)
Supplement: Supplementary file 5 [file 813FileS1.pptx]

## Slide 1
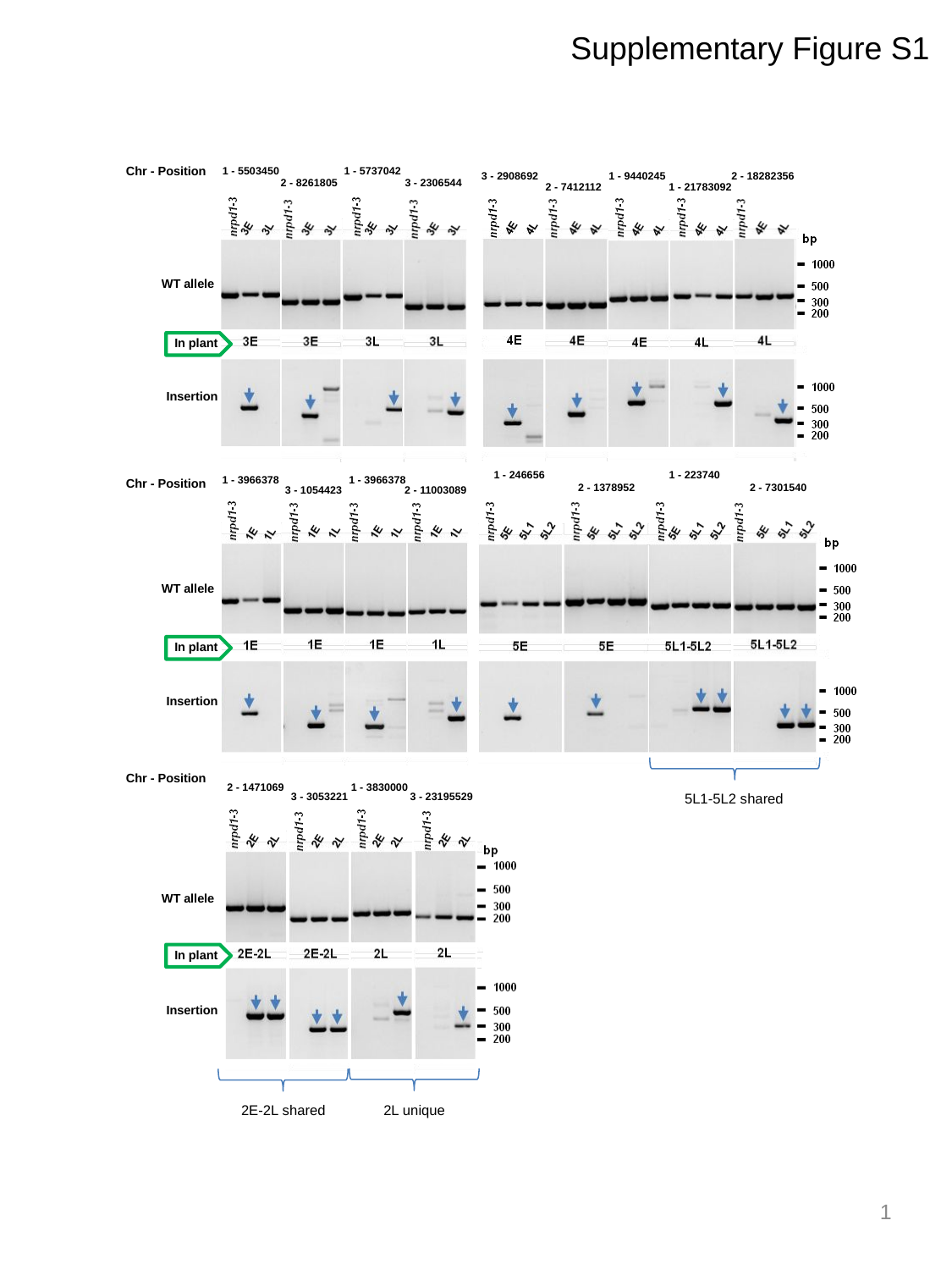

Supplementary Figure S1
Chr - Position
1 - 5503450
1 - 5737042
3 - 2908692
1 - 9440245
2 - 18282356
2 - 8261805
3 - 2306544
2 - 7412112
1 - 21783092
WT allele
In plant
Insertion
1 - 246656
1 - 223740
1 - 3966378
1 - 3966378
Chr - Position
2 - 1378952
2 - 7301540
3 - 1054423
2 - 11003089
WT allele
In plant
Insertion
Chr - Position
2 - 1471069
1 - 3830000
3 - 3053221
3 - 23195529
WT allele
In plant
Insertion
2E-2L shared
2L unique
5L1-5L2 shared
1

## Slide 2
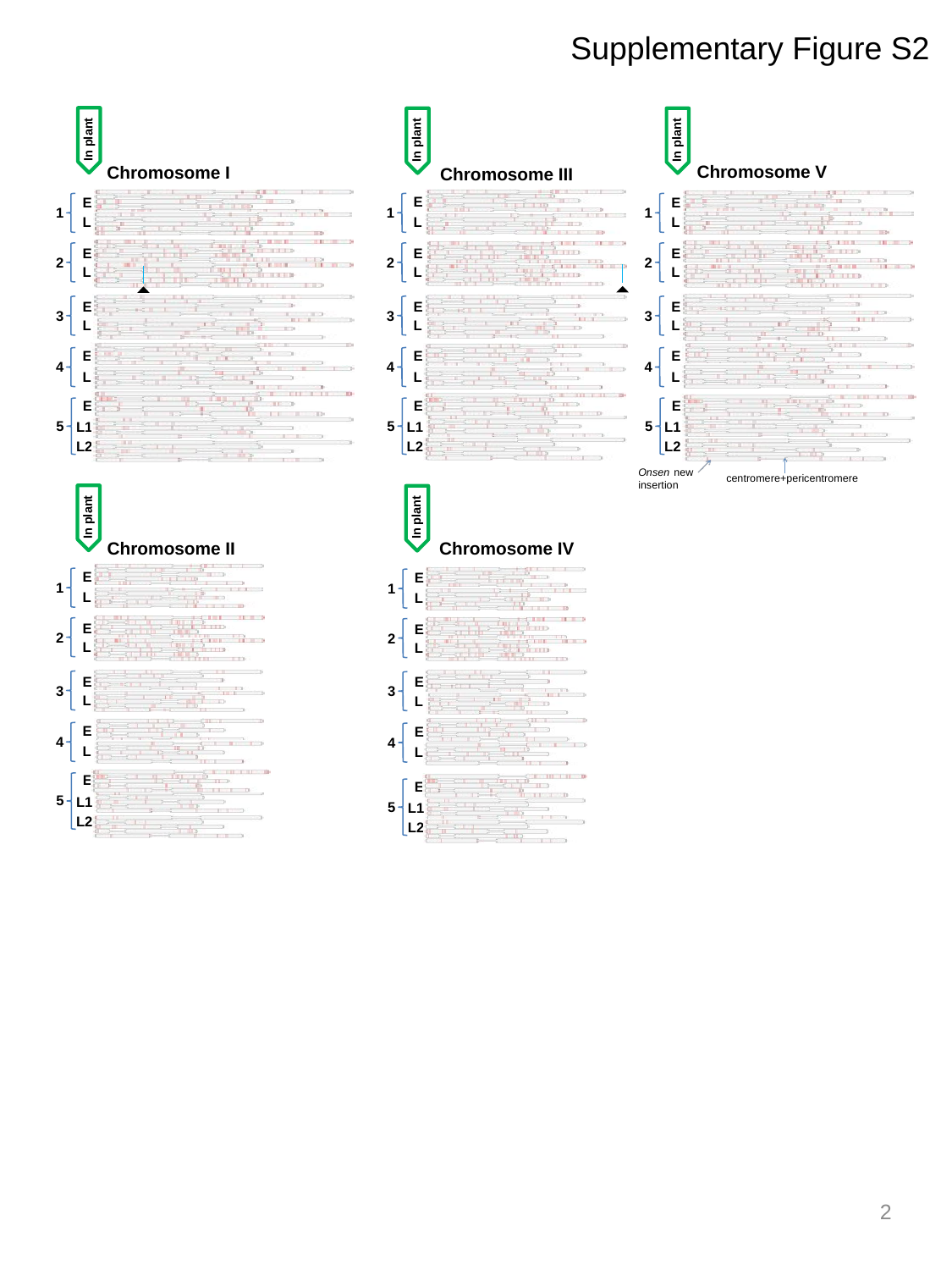

Supplementary Figure S2
In plant
In plant
In plant
Chromosome V
Chromosome I
Chromosome III
E
E
E
1
1
1
L
L
L
E
E
E
2
2
2
L
L
L
E
E
E
3
3
3
L
L
L
E
E
E
4
4
4
L
L
L
E
E
E
5
5
5
L1
L1
L1
L2
L2
L2
Onsen new insertion
centromere+pericentromere
In plant
In plant
Chromosome II
Chromosome IV
E
E
1
1
L
L
E
E
2
2
L
L
E
E
3
3
L
L
E
E
4
4
L
L
E
E
5
L1
5
L1
L2
L2
2

## Slide 3
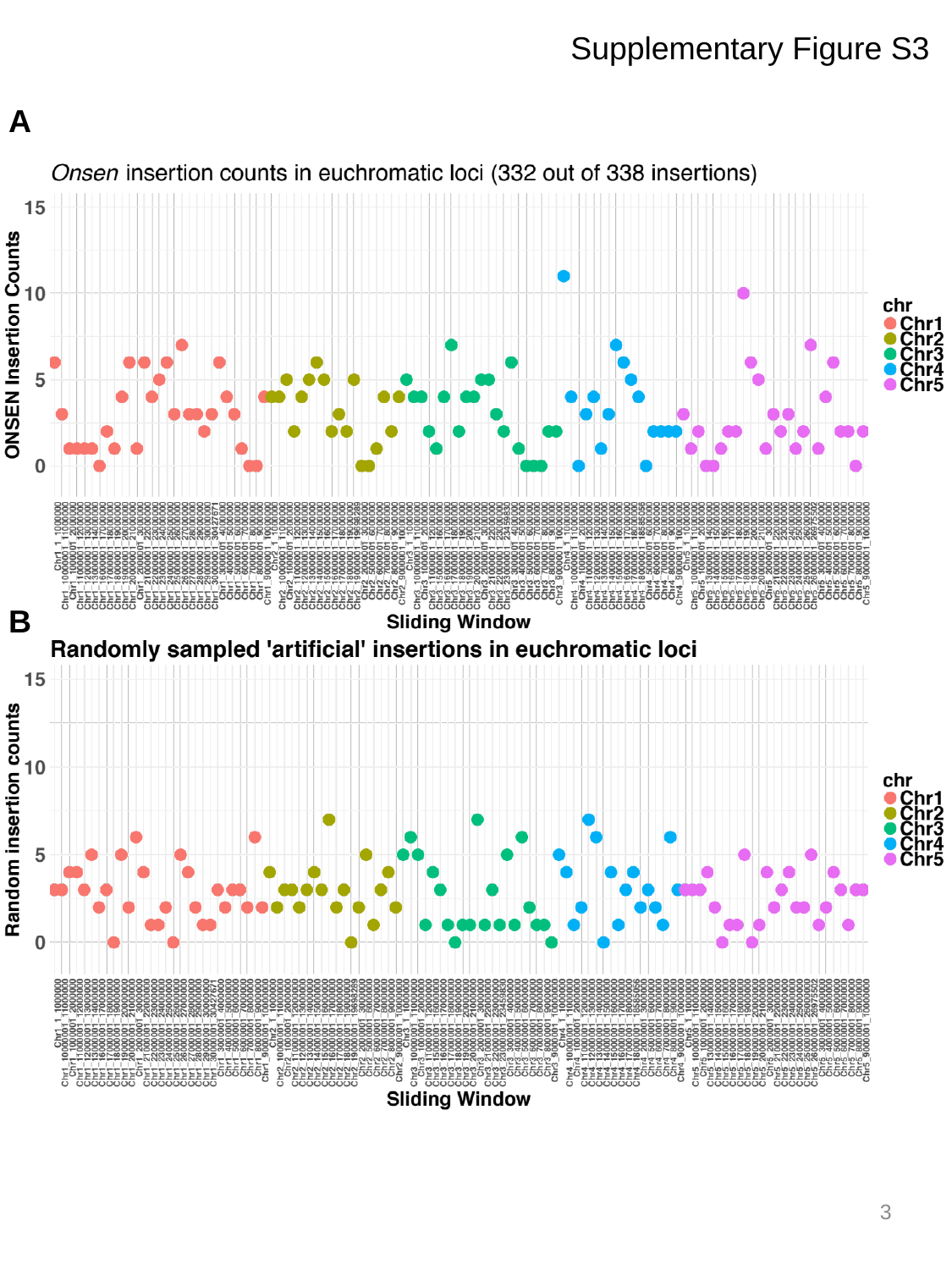

Supplementary Figure S3
A
B
3

## Slide 4
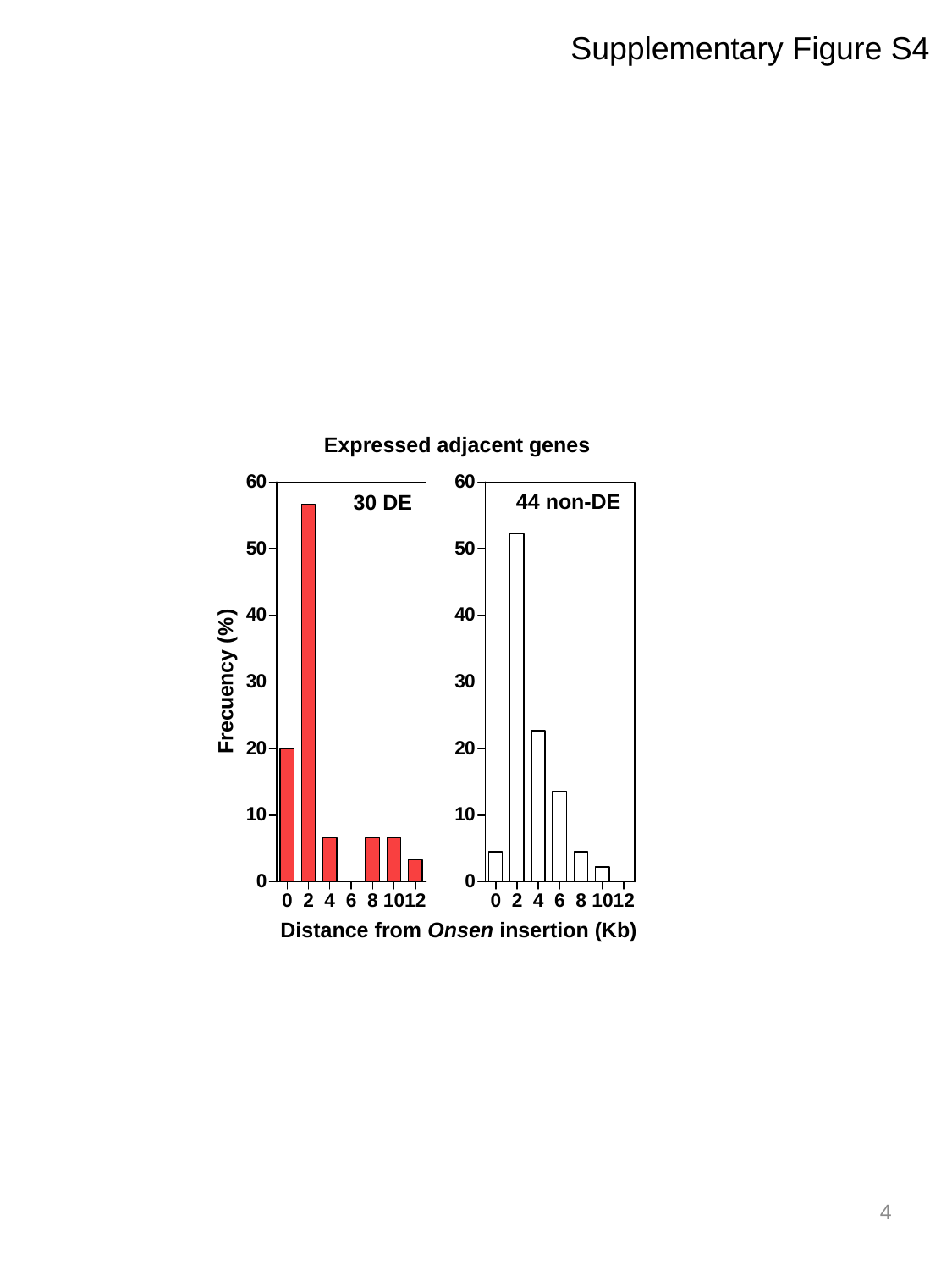

Supplementary Figure S4
Expressed adjacent genes
44 non-DE
30 DE
Distance from Onsen insertion (Kb)
4

## Slide 5
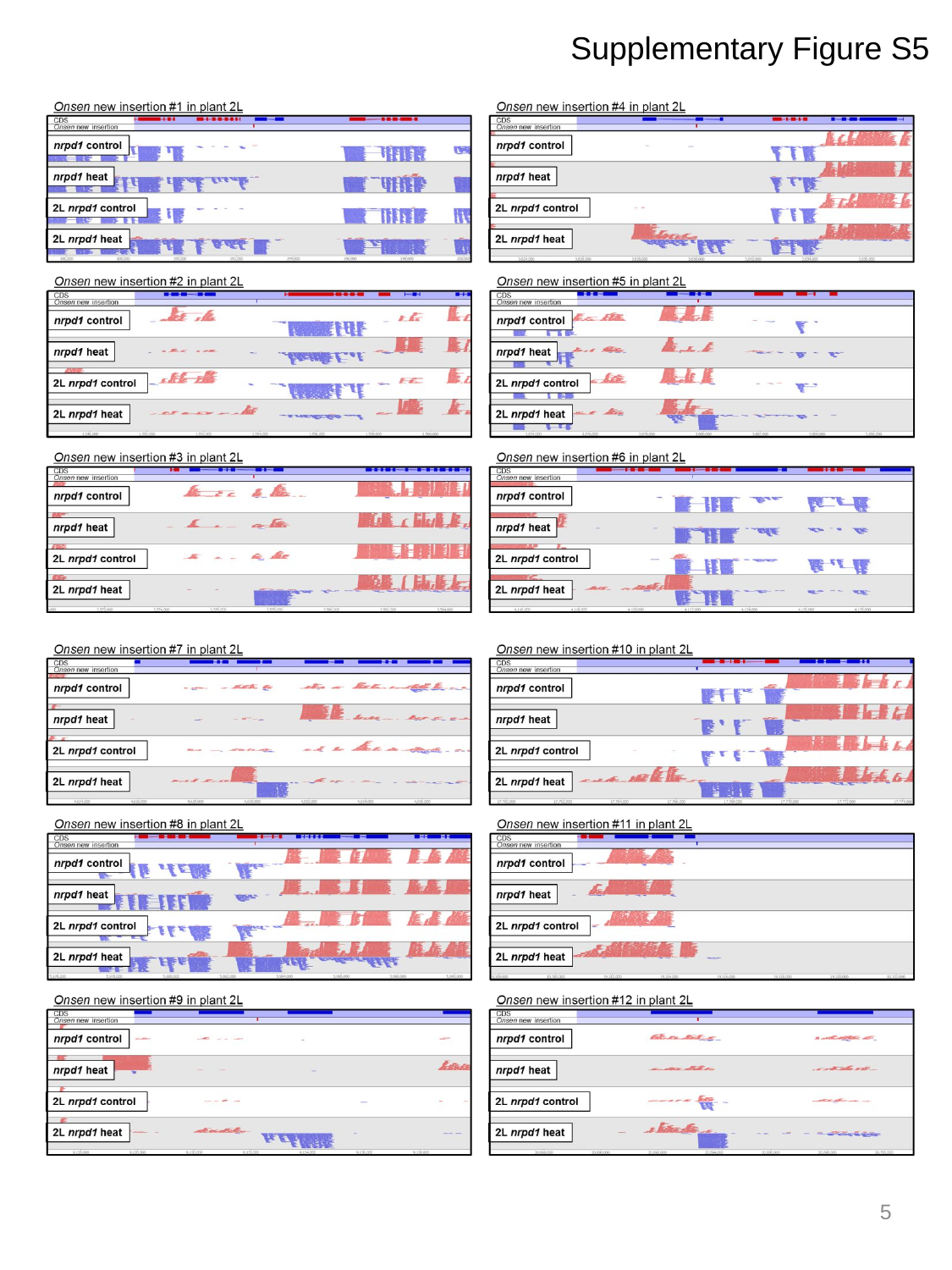

Supplementary Figure S5
5

## Slide 6
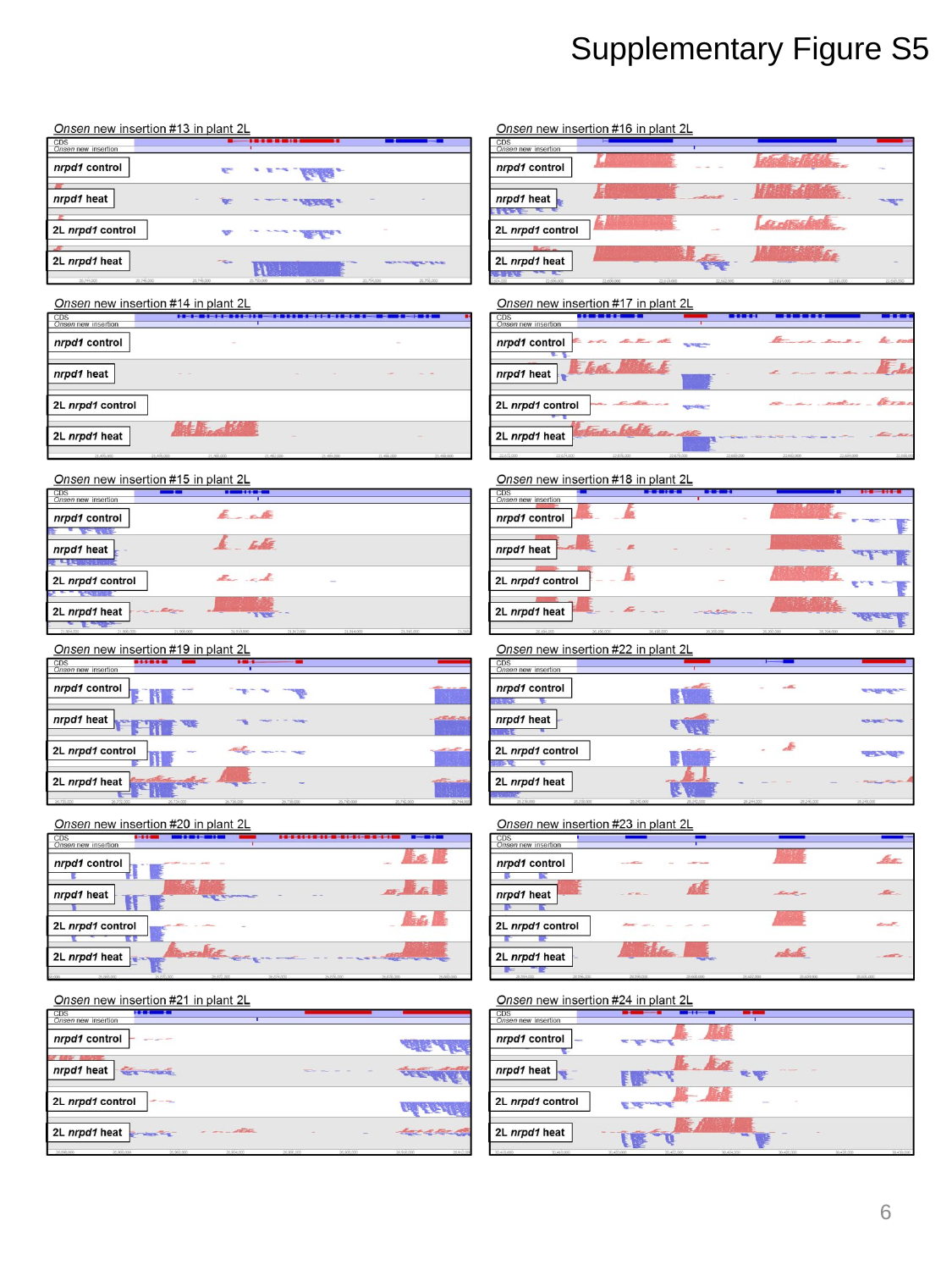

Supplementary Figure S5
6

## Slide 7
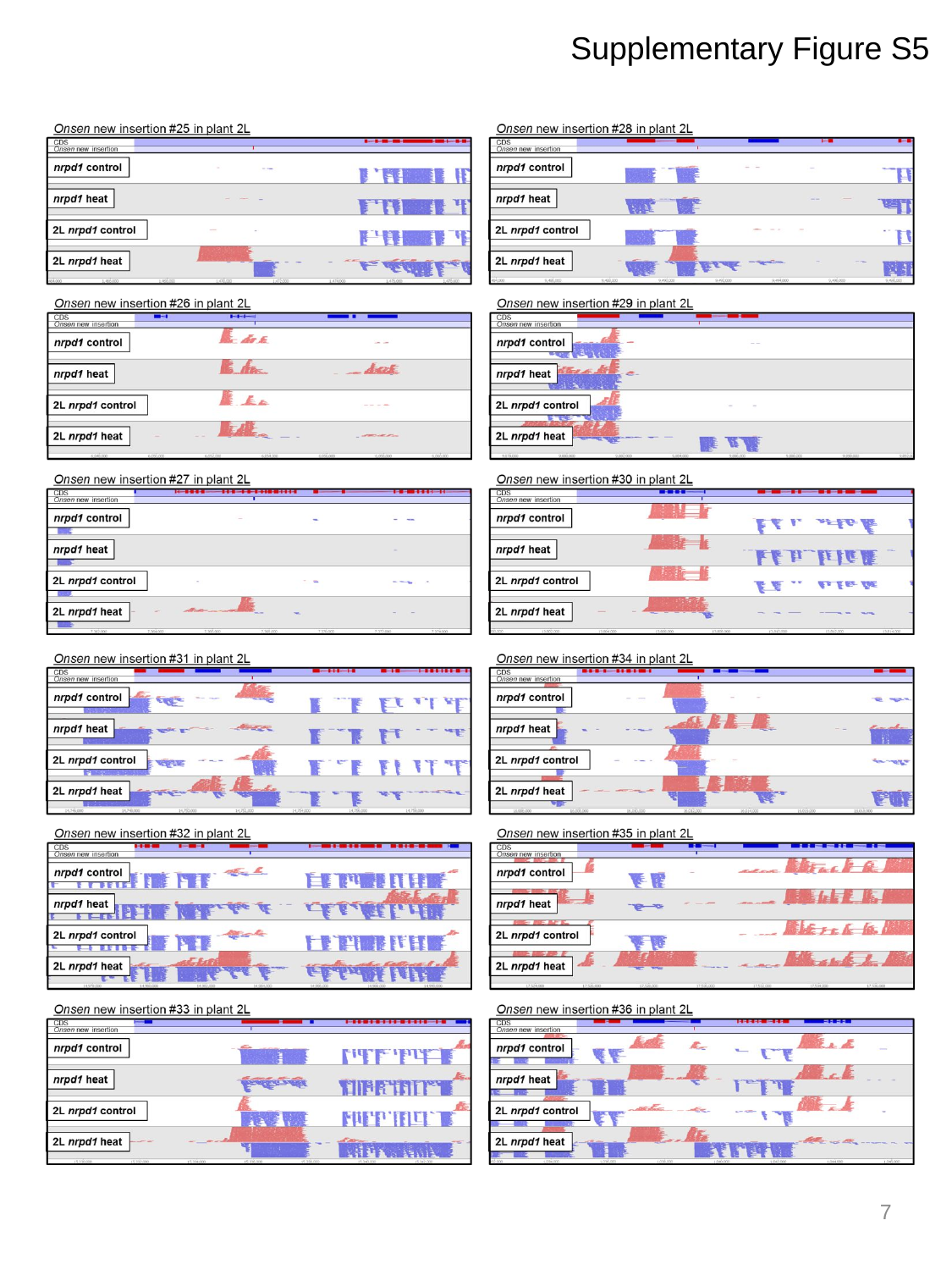

Supplementary Figure S5
7

## Slide 8
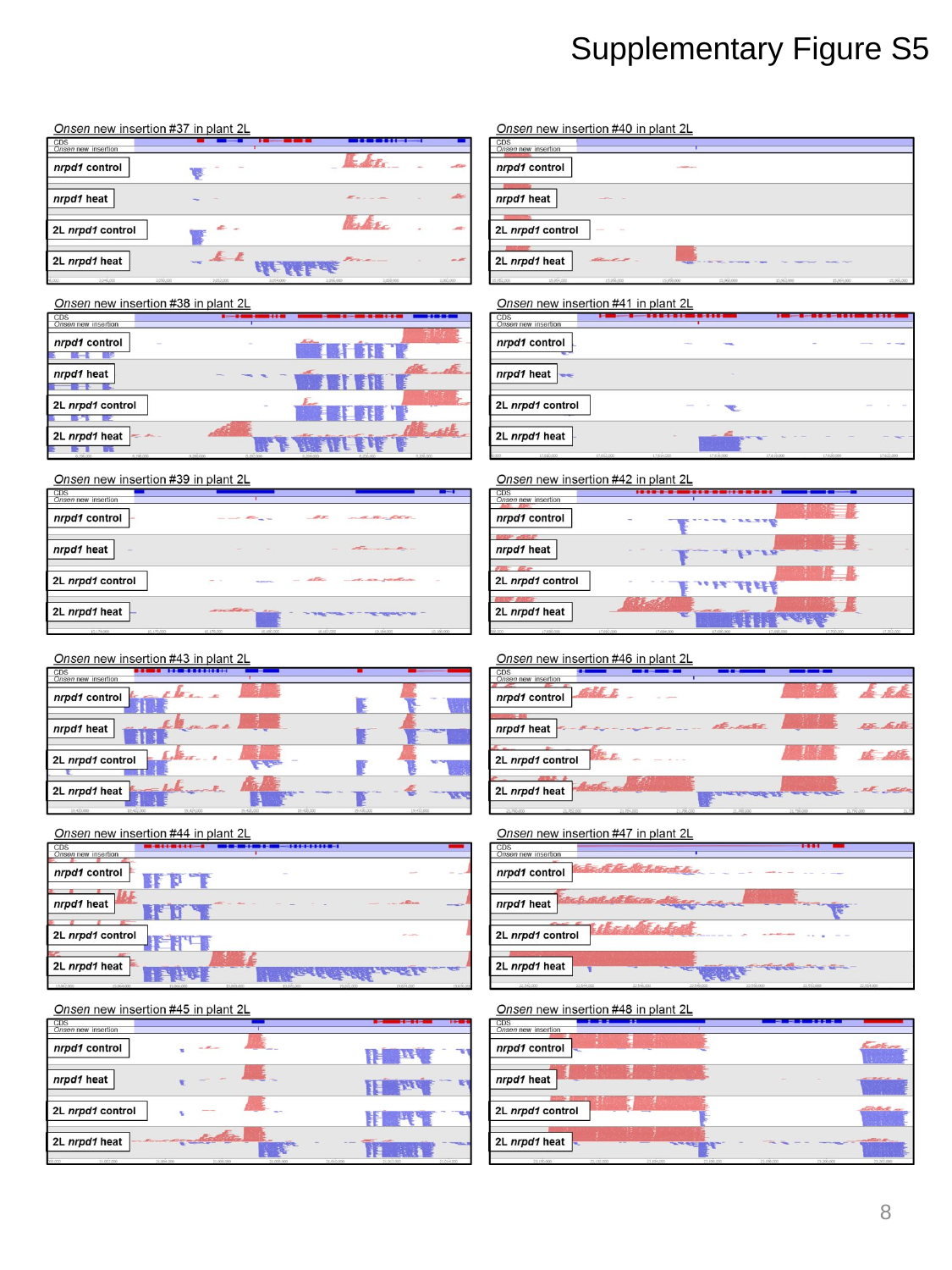

Supplementary Figure S5
8

## Slide 9
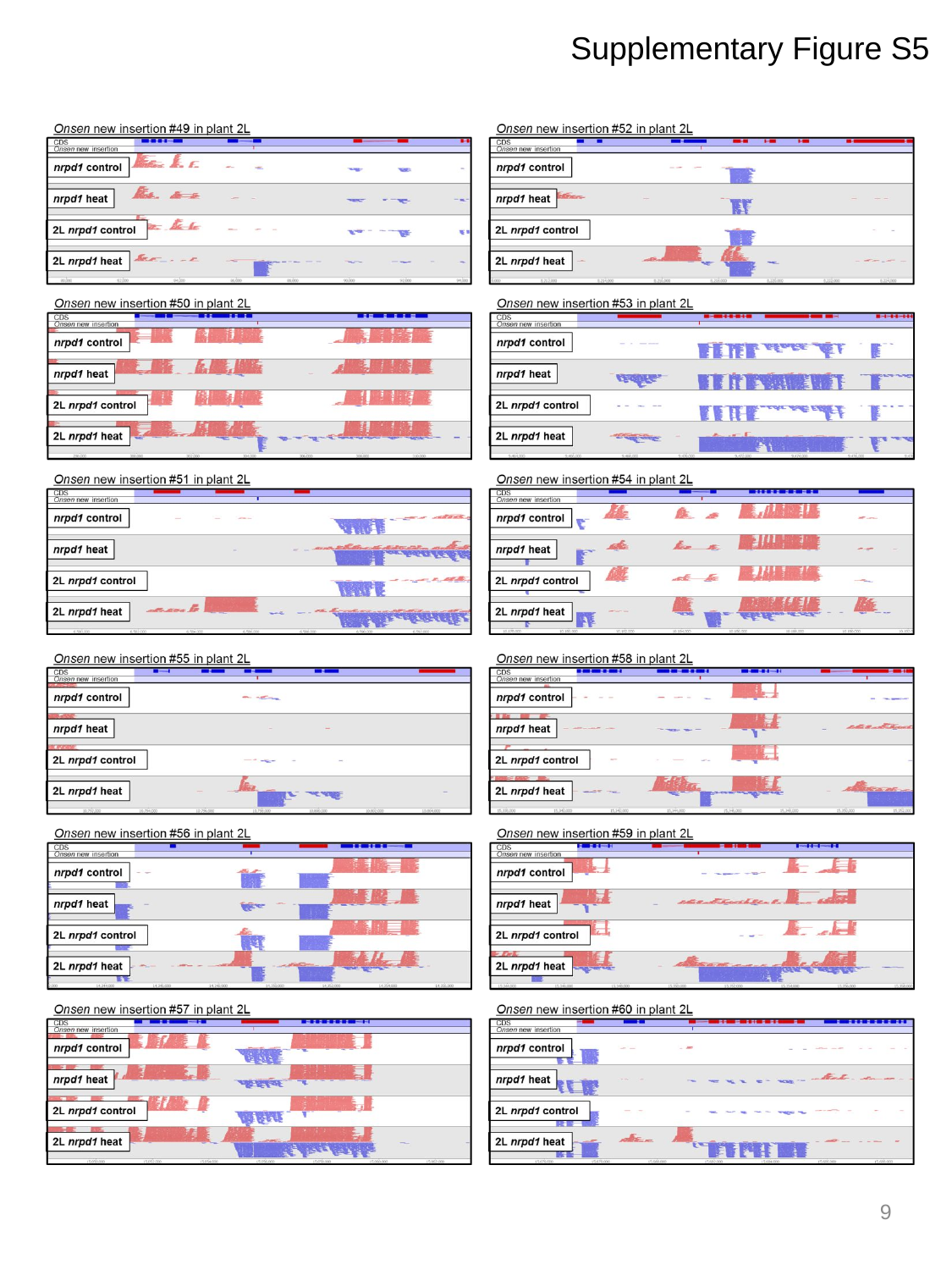

Supplementary Figure S5
9

## Slide 10
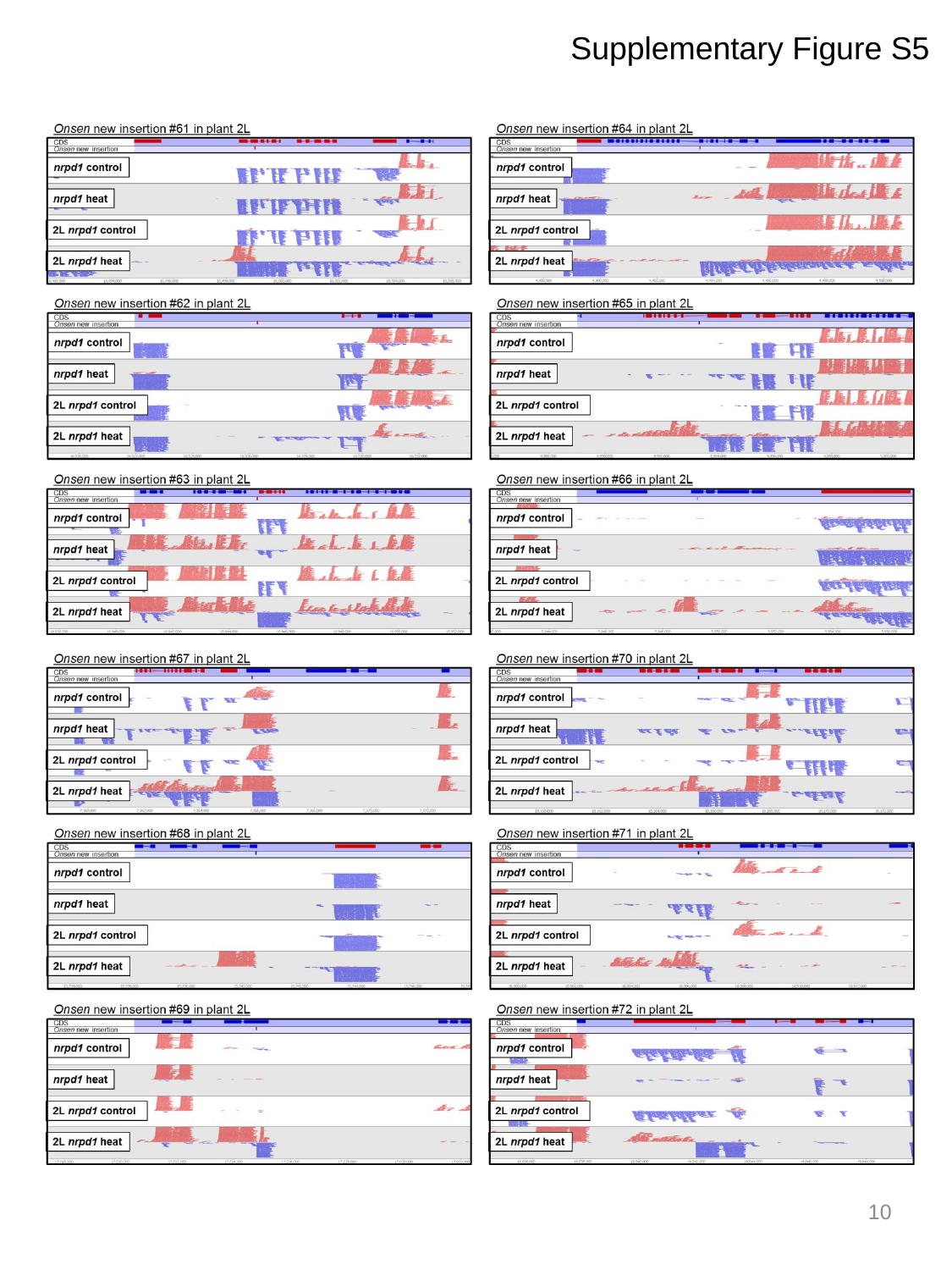

Supplementary Figure S5
10

## Slide 11
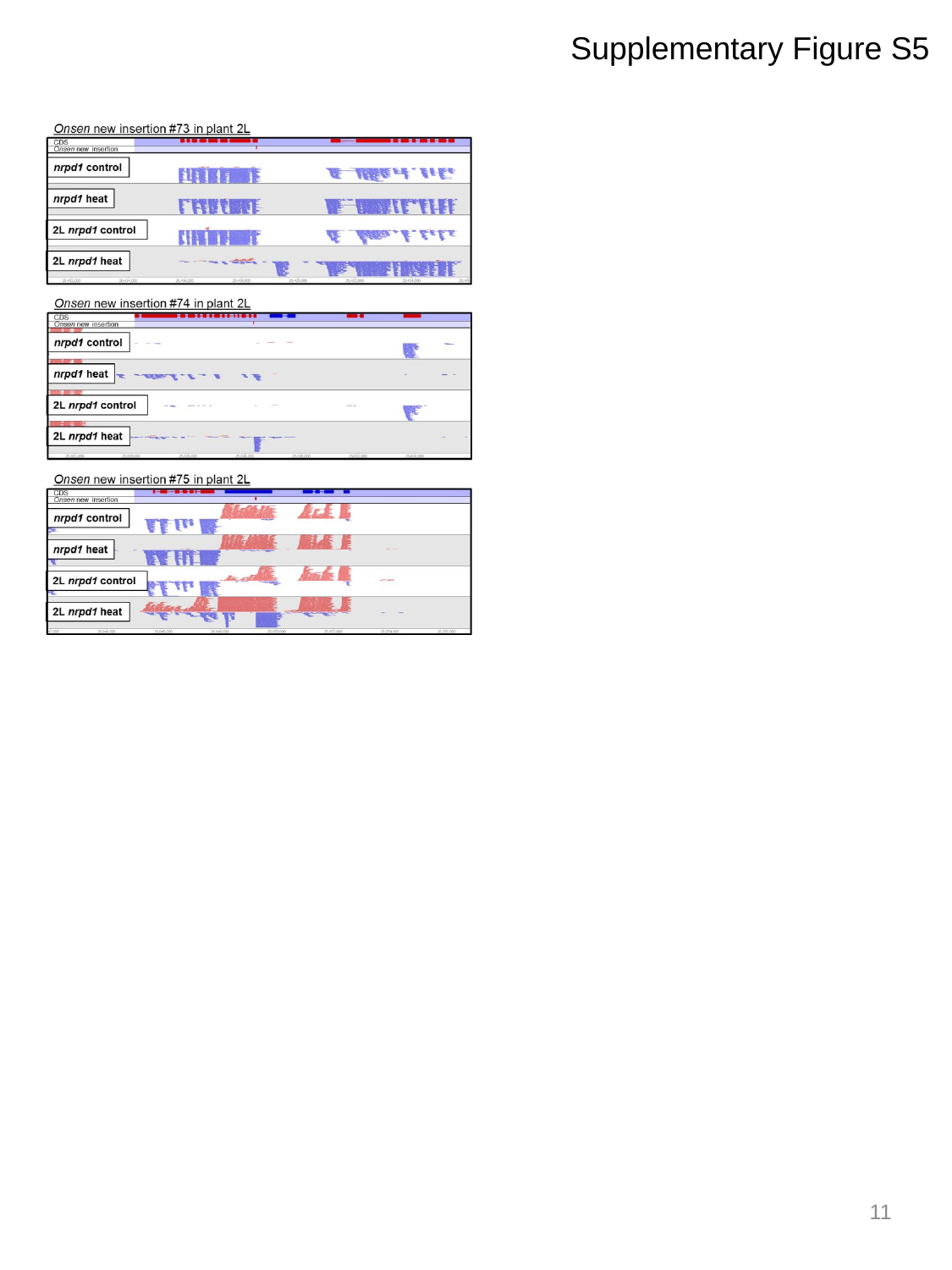

Supplementary Figure S5
11
